# The Impact of Toothbrushing on Oral Health, Gingival Recession, and Tooth Wear—A Narrative Review

**DOI:** 10.3390/healthcare13101138

**Published:** 2025-05-14

**Authors:** Santhosh Kumar, Pratibha Gopalkrishna, Ayman K. Syed, Abishikka Sathiyabalan

**Affiliations:** 1 Department of Periodontology, Manipal College of Dental Sciences, Manipal, Manipal Academy of Higher Education, Manipal 576104, Karnataka, India; 2 Manipal College of Dental Sciences, Manipal, Manipal Academy of Higher Education, Manipal 576104, Karnataka, India; ayman36@gmail.com (A.K.S.); a.sathiyabalan@hotmail.com (A.S.)

**Keywords:** bristle diameter, brushing force, cervical abrasion, gingival abrasion, gingival recession, toothbrush

## Abstract

**Background/Objectives**: Toothbrushing is a recommended daily practice that helps sustain oral health. However, if performed improperly, it can lead to loss of tooth structure and injury to soft tissues. We explored this topic with an extensive literature search. **Methods**: A literature search was performed across textbooks and journals for original research and review articles in Scopus, PubMed, PubMed Central, and Cochrane databases, published between 1967 and 2024. **Results**: The search result yielded 118 articles that were suitable to include in this review. Toothpaste abrasivity plays a major role in combination with toothbrush forces. Therefore, maintaining forces between 2 and 3 N may be gentler on the tissue. Electric toothbrushes are safer. Toothpastes with low RDA values are also less abrasive. Active ingredients in whitening and desensitizing toothpaste can induce tooth wear. Remineralizing agents have the potential to manage the associated lesions. **Conclusions**: Cervical abrasions and gingival recession occur frequently due to oral hygiene measures. Standards in oral hygiene aid to match patient needs can prevent hard and soft tissue loss.

## 1. Introduction

Toothbrushing is an oral hygiene practice that dates back many years and has successfully maintained oral health. Past oral hygiene practices have utilized twigs, fingers, and rough cloth. The toothbrush design we use today, with nylon bristles and a plastic handle, was accepted by the 1930s due to its ease of use and cost-effectiveness, promoting daily brushing habits. However, brushing can also be detrimental for the individual if not performed correctly. Oral hygiene practices using tooth powders and tree sticks increased gingival recession, gingival bleeding, and excessive tooth wear and were inferior in plaque control compared to brushes [1]. Tooth wear is further aggravated by an individual’s brushing style or the presence of incorporated abrasives and other medicaments, although these cannot be verified [2].

As recommended by various researchers, good oral hygiene maintenance requires approximately two minutes of brushing [3]. Many individuals tend not to follow these recommendations, mainly due to a lack of knowledge and dexterity; hence, they perform vigorous toothbrushing for relatively short durations, resulting in cervical abrasions with recession of the associated gingival margins. Non-carious cervical lesions arise from abrasive action due to improper brushing force, technique, and duration [4]. Glorified advertising to promote abrasive toothpaste can persuade the general population to buy these products. This review paper explores the influence of toothbrushing and related factors on the changes in oral hard and soft tissues.

## 2. Methodology

A literature search across textbooks, journals, and review articles in Scopus, PubMed, PubMed Central, and Cochrane databases identified contributory factors for tooth/soft tissue abrasion. The search terminologies included toothbrush, toothpaste, dentifrices, toothbrush filaments, toothbrush bristles, toothbrush handle, brushing force, tooth abrasion, gingival abrasion, and gingival recession. We did not restrict the time frame of the search to understand the evolution of information relevant to the review from 1967 to 2024.

The inclusion criteria for article selection followed the aim of this review. We excluded articles that were not indexed and in languages other than English. We included about 118 articles in this review, which were scrutinized by all the authors based on the toothbrush parts, design, filaments, material used, force applied, toothpaste, and gingival/tooth abrasion.

## 3. Toothbrushing Effects on Hard/Soft Issue

The general belief is that forceful and increased pressure during brushing leads to better plaque removal. Overaggressive or abrasive brushing, which entails brushing with a medium/hard toothbrush, highly abrasive toothpaste, heavy toothbrushing forces (>3 N), and incorrect brushing techniques can cause either cervical abrasions or gingival recession, eventually leading to dentinal hypersensitivity. Cervical abrasions affect over 70% of the population with extreme distress, discomfort, and pain [5]. Toothbrush-related abrasive lesions are usually wedge-shaped, with the initial loss of enamel in the cervical region of the crown, progressing to deteriorate the dentin and extending to the pulp and devitalizing it.

Available treatment modalities for cervical abrasions include defect restoration using tooth-colored restorative materials such as composite or glass ionomer cement, which physically occlude the dentinal tubules, decreasing patient complaints of sensitivity. Continual aggressive brushing causes gradual restoration wear, and the subsequent pulpal pathology indicates invasive treatment options such as root canal treatment and extraction.

A receding gingival margin is another consequence of forceful brushing. An epidemiological study determined a 40.98% gingival recession rate among 710 subjects. Multiple factors were related to this condition [6]. Root surface exposure increases susceptibility to caries and predisposes the teeth to erosion or cemental abrasion. Further, interproximal recession creates difficulty in oral hygiene maintenance.

## 4. Patient-Related Factors Associated with Tooth Wear

### 4.1. Brushing Force

Apart from the emphasis on oral hygiene aids, an individual’s dexterity also plays a role in effective oral hygiene practices. Although it has been proposed that a brushing force of approximately 1N should be maintained [7], the observed mean brushing force was found to be 2.3 ± 0.7 N (Max 4.1 N) [8]. Increased brushing force does not enhance plaque removal efficiency. Gentle brushing is recommended, with forces between 2.5 N–3 N [4]. However, remaining within this optimal range can be difficult [9].

Additionally, brushing forces vary with the brushing cycle [10]. Difficult access to the lingual and palatal sites leads to unconscious increases in the brushing force applied, unlike in the vestibular areas (Table 1). Due to dominant hand dexterity, an individual is likely to apply more force on the opposite arch while brushing than on the non-dominant side. A previous study on adult toothbrushing habits observed that 12.6% of participants brushed with forces of 1.5 N or less, and 17.5% brushed with a force of 3 N or greater, with males having a higher brushing force than women.

**Figure 1:**
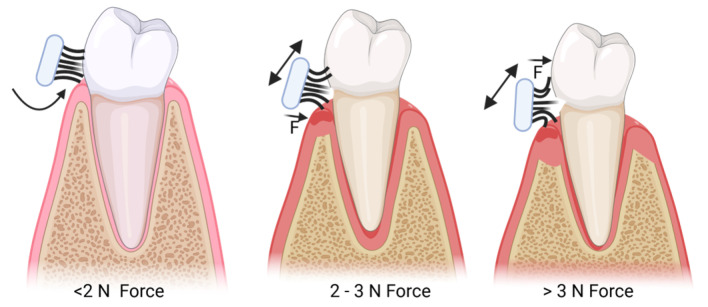
Brushing technique changes with increased brushing force enhancing wear.

Studies show a clinical correlation between force and abrasion/gingival recession. Abrasive lesions corresponded to brushing forces of 2.9 ± 0.4 N, unlike those devoid of abrasion, where the brushing forces were lower, 2.1 ± 0.3 N. Further, severe recession resulted from forces of 3.8 ± 0.5 N, minor recession from 2.4 ± 0.4 N, and no recession from 2.1 ± 0.3 N of force [11] (Figure 1). Applying the optimal brushing force is difficult since we generally do not monitor it.

Hard manual brushes increase pressure considerably [12,13]. To overcome this issue, innovations such as pressure-detecting electric toothbrushes have been made available in the market. Pressure-detecting mechanisms self-correct the pressure applied to the tooth. An earlier study showed that pressure detection improved the amount of brushing force transferred to the tooth, with relatively thorough plaque debridement in the long term [14]. Heasman et al. observed that with pressure-controlled electric toothbrushes, the brushing forces decreased within 6 weeks of training. The incorporated click mechanism provided feedback to achieve the optimal brushing force [15]. The Rotadent^®^ toothbrush (Zila, Inc., Phoenix, AZ, USA) reduces abrasion due to its low brushing pressure. It also requires very little toothpaste [11,16]. Studies have suggested that electric toothbrushes are safer and induce less tooth surface loss [4,17,18] (Table 1). On the flip side, electric toothbrushes are expensive.

**Table 1:** Summary of studies on the force of brushing.

Author and Year	Aim	Study Type, Sample Size, Pressure/Force	Outcome
Souza C et al. (2021) [13]	Manual toothbrushes’ brushing loads on the progression of erosive tooth wear (ETW) on enamel.	In vitro 60 bovine incisors allocated into 6 groups and divided into 6 different toothbrushes. Brushing load of 3 N and 1.5 N forces.	Hard brushes are not recommended for use by patients with erosive tooth wear.
Bizhang M et al. (2017) [19]	Susceptibility of dentin to brushing abrasion using four different toothbrushes with the same brushing forces.	In vitro 72 impacted third molars; rotating–oscillating, sonic, and two types of manual toothbrushes. Brushing force was set to 2 N (260 min of brushing).	Manual toothbrushes are significantly less abrasive compared to powered toothbrushes in a 8.5-year simulation.
Rosema et al. (2014) [18]	Manual toothbrush and an oscillating–rotating toothbrush. Compare groups in terms of both the level of existing gingival recession and the extent of gingival abrasion before and after a single toothbrushing exercise.	Cross-sectional Uncontrolled epidemiological study with 181 participants ranging from 18 to 35 years.	The powered toothbrush is as safe as a manual one. The force exerted by the powered toothbrush is lower than that of the manual one.
Wiegand A et al. (2013) [4]	Determine and compare the brushing forces with manual and sonic toothbrushes; brushing forces on abrasion of sound and eroded enamel and dentin.	In vivo 27 Volunteers (5 males and 22 females; 18–55 Years); one manual and two sonic toothbrushes.	Manual toothbrush, 1.6 ± 0.3 N; sonic toothbrush, 0.9 ± 0.2 N. The manual toothbrush caused the highest abrasion of sound and eroded dentin; patients with severe tooth wear and exposed (eroded) dentin surfaces should use sonic toothbrushes.

### 4.2. Brushing Technique

The ideal brushing technique suitable for an individual differs according to the condition of the oral cavity and the individual’s dexterity. The horizontal scrub technique, most commonly used, promotes cervical abrasions. The repetitive back-and-forth bristle motion over the gingiva contributes to receding gingival margins [20]. The Bass is the most effective in removing the biofilm within the sulcus and is thus widely recommended [21]. Leonard’s method is easy for children to adapt interproximally. Stillman’s method facilitates plaque removal from exposed gingival embrasures in patients with severe gingival recession. Charter’s method finds favor in the case of fixed-appliance treatments or post-periodontal surgery for its ability to direct debris away from the gingival margins and thoroughly clean the interdental regions. Fone’s method is a circular brushing technique used for young children and differently abled individuals due to their lack of dexterity [22].

Regardless of the technique, toothbrushing duration should persist for 2–3 min, with 30 s per quadrant. Adopting individually suited brushing methods for the required duration is difficult without adequate supervision. A previous study noted that although the participants received training on the proper brushing technique for the study duration, their old brushing practices could not be amended [9]. Incorrect brushing techniques can precipitate abrasive tooth wear and root exposure with time.

Padbury (1974) [23] published his findings on abrasion caused by different toothbrushing techniques. He concluded that the scrubbing method creates more localized abrasions in the cervical region than the roll technique, where the entire crown surface is abraded [23]. However, Muller-Bolla and Courson (2013) studied children’s brushing ability to remove dental plaque, finding that the horizontal brushing method was the most effective in the ages 6–7 [24]. Dental professionals must look into their patients’ hand-skill motion and oral condition before advising any brushing method. Patients should be encouraged to practice the appropriate technique for the optimal duration.

## 5. Toothbrush-Related Factors Influencing Tooth Surface Wear

### 5.1. Toothbrush

**Figure 2:**
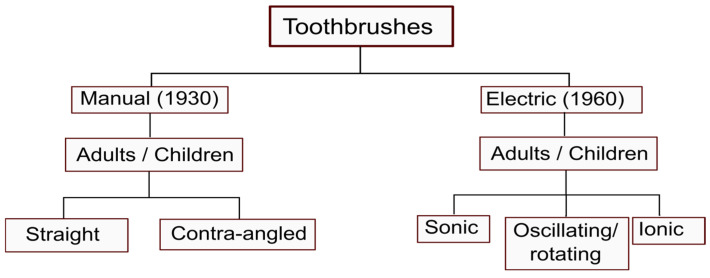
Different types of toothbrushes.

A toothbrush is an oral hygiene instrument used to clean the oral cavity. It consists of a brush head with tightly packed bristles in tufts and a handle enabling individuals to access hard-to-reach areas. With the advancements of science, there have been many modifications to the design of the average manual toothbrush, including changes to the bristle tuft arrangement, filament characteristics, number of filaments or tufts, length of the bristles, and handle size (Figure 2). Further, electric toothbrushes, first introduced in 1960, include sonic, oscillating–rotating, and ionic varieties. These modifications can be beneficial as well as harmful.

### 5.2. Handle

Manual toothbrushes started with handles made of bones or bamboo in the early 1800s. These were hard materials that did not absorb heavy brushing forces. It was in the 1900s when celluloid handles replaced bone and bamboo handles. Present toothbrush handles are moldable, recyclable thermoplastic materials known as polypropylene and polyethene [25]. The material is flexible, weatherproof, easy to process, and less costly, with good chemical resistance. Polypropylene is less sturdy but takes less time to biodegrade than polyethene. It is highly fatigue-resistant and can withstand continuous flexing. Other materials, such as rubber, are added to provide consumers with variety.

Toothbrushes with handles made of a homogenous material are likely to absorb a constant amount of force compared to a toothbrush handle made of various materials. Hence, with toothbrush handles made of polyethene and additional materials, excessive forces exerted by the individual will be absorbed, averting over-brushing.

### 5.3. Bristles (Filaments)

The bristles of a toothbrush are made of nylon (Wytex 6.12S) and sometimes polybutylene terephthalate (PTB). The PTB filament has good bend recovery and stiffness when wet, as well as a long shelf life [26].

Manual toothbrushes can be ultra soft, soft, medium, and hard based on bristle diameter (Table 2). Thinner bristles are gentle on teeth, unlike thicker, hard bristles [27]. Stiffer bristles dislodge deposits, and patients brush better with broader-diameter bristles. Plaque removal near the gingival margin is better with a bristle diameter of 0.2 mm than 0.18 mm [4,27]. A hard-bristled brush produces more tooth surface loss than softer bristles with the same average force [11,28].

Manual toothbrushes vary according to the arrangement of bristle tufts. The bristle profile can be a flat rippled castle, a multilevel castle with flared bristles, a multilevel/mono tip, extended bristles, bi-level flex bristles, or angled bristles. These variations exist to enhance plaque removal from hard-to-reach areas. Angled bristles are 12–15% more effective in cleaning interproximal regions. Multilevel bristle tufts have an 8–9% higher cleaning efficiency, as their varying levels allow the tufts to act independently. Longer bristles reach farther between the teeth compared to the conventional flat-trim toothbrush. Tightly packed bristle tufts block individual tufts from reaching interproximal areas [21]. Cross-angled/multilevel/flex heads are less abrasive [29].

Toothbrush efficiency also depends on the stiffness and end shape of the brushes. Flat-trim toothbrush bristles cause the least amount of surface abrasion [30]. Pereira et al. 2023 [31] found bristle roundedness caused more loss of dentin than enamel. Reports also suggest that soft-bristled toothbrushes resulted in more wear than hard-bristled ones [32]. Soft-bristled toothbrushes can maintain contact between the toothpaste and the tooth surface for much longer [28]. The bristles are more flexible, accessing a greater surface area than hard-bristled toothbrushes [33]. Therefore, hard-bristled brushes are not for patients with erosive tooth wear.

Toothpaste tends to influence bristle wear. Extra-soft bristles with abrasive toothpaste (containing calcium carbonate, calcium bicarbonate, and silica) induce significant changes in bristle morphology, accelerating toothbrush aging [34]. Rounded or tapered end bristles have different effects on the gingiva [35,36,37,38,39,40,41]. A systematic review [42] concluded that soft and extra-soft bristles were safe. ’Medium-hard’ bristles seem to induce gingival fissures [43]. Others have suggested that end-roundedness does not have clinical effects on gingival tissue.

**Table 2:** Different bristle types based on the diameter of the bristles.

Bristle Type	Diameter	Authors
Ultrasoft	0.09–0.11 mm	Souza C [13]
Soft	0.15–0.2 mm	Burgett F, Souza C, Fraleigh C, Bizhang M [10,12,13,17]
Medium	0.18–0.19 mm	Fraleigh C, Souza C [13,32]
Hard	0.20–0.30 mm	Burgett F, Fraleigh C Souza C [10,13,32]

### 5.4. Electric vs. Manual Toothbrushes

Electric toothbrushes were first developed in Switzerland in 1939 [44], with varied designs and bristle motions like rotation, oscillation, sonic movement, or ionic movement, hoping to address the disparities in individual brushing practices, techniques, and durations; studies on electric brushes showed dental plaque and gingivitis reduction with no soft and hard tissue damage, owing to the lower brushing forces, providing an option for patients with gingival recession [45,46,47]. Yet, studies suggest that an electric toothbrush creates brushing forces ranging from 0.5 N to 6 N; therefore, tooth structure loss and gingival recession are likely if the individual applies the same force as with a manual toothbrush [4]. Salzer reported no difference in plaque control between manual and electric toothbrushes in patients with gingival recession [48].

A study conducted over eight and a half years showed the mean tooth surface loss with different types of toothbrushes to be 21.03 µm of loss with a sonic toothbrush, 15.71 µm with an oscillating–rotating electric toothbrush, 6.13 µm with a flat-trim manual toothbrush, and 2.50 µm with a ripple-shaped manual toothbrush [19]; consequently, it is clear that electric toothbrushes are more abrasive than manual toothbrushes when brushed with the same force and duration. Combining toothpaste with a sonic toothbrush increases the chances of abrasion, depending on the abrasive particle size [49]. A survey by Heasman was inconclusive about the association between toothbrushing and non-carious cervical lesions [50].

### 5.5. Toothpastes

Toothpaste offers enhanced esthetics in maintaining oral hygiene. It consists of fluoride (anti-cariogenic), glycerol, sorbitol (humectant), calcium carbonate (abrasive agent), sodium lauryl sulfate (surfactant), flavoring agents, and water (solvent). Additional therapeutic/cosmetic ingredients incorporated are peroxide, diamond particles, charcoal (for whitening), strontium chloride, sodium fluoride, calcium carbonate, and potassium nitrate (for desensitization). They have been classified based on certain characteristics of their chemical composition [51].

Studies have reported an increased occurrence of cervical abrasion when brushing with toothpaste. Salivary pH and combined chemical and mechanical stresses also influence the extent of tooth wear. Surface roughness and wear depth increase when erosion adds to tooth abrasion [52]. Citric acid was more abrasive than hydrogen peroxide [53,54].

Toothpaste abrasivity is determined by the Relative Enamel Abrasivity (REA) and Relative Dentin Abrasivity (RDA) indexes or Ra and Rz values using a profilometer [55]. According to the American Dental Association, RDA values of 0–70 are minimally abrasive, values of 71–100 are moderately abrasive, values of 101–150 are highly abrasive, and values of 151–250 are harmful [56]. The modified surface microhardness measurement can also determine surface roughness [57]. A toothpaste with low REA and RDA values should be considered when selecting toothpaste [58].

The brushing movement, combination of the dilution ratio, diluent of the toothpaste slurry, particle size, and toothpaste ingredient fraction affect the progression of enamel surface loss [59,60,61]. While hydrated silica in toothpaste (moderately abrasive) provides higher cleaning efficiency [62], increasing the silica percentage increases the abrasive value [63]. Baking soda can remove stains and is therefore abrasive, but the abrasivity is very low [64]. Similarly, brushing with fluoride-containing toothpaste alters the surface enamel [65], but there are contradictory reports. Sodium hexametaphosphate increases erosive tooth wear and decreases the anti-erosive effects of fluoride and tin [66]. The addition of diamond particles/powder showed higher abrasive wear of enamel [67] than dentin [68]. REA and the RDA values are higher in toothpaste containing diamond particles, and therefore, such pastes should be cautiously prescribed [58]. Toothpaste containing bioactive glass can reduce enamel loss and potentially remineralize the enamel after acid exposure [69]. Adding hydroxyapatite crystals in toothpaste can have more erosive effects on enamel than remineralization [70].

The toothpaste slurry and filament stiffness also influence toothbrush abrasion. Abrasive slurries result in non-carious cervical lesions with medium or high toothbrush stiffness [4,71,72]. Hence, brushing with minimally abrasive toothpaste is advisable in such patients [73]. Particle size increases also compound abrasive effects, as observed with 45S5 bioactive-based toothpastes, particularly intensified at the dentin junction [74].

Tooth wear from fruit juices is significant due to their acidic content [75]. In an in vitro study, exposure of enamel to orange juice or citric acid for 3 min created a softened enamel. Subsequent brushing of this surface with artificial saliva had minimal effect on remineralization, but incorporating a toothpaste enhanced enamel abrasion [76,77]. Therefore, brushing the surface of eroded enamel aggravates abrasive effects compared to the surface of sound enamel [78]. Further, there was remineralization of acid-etched enamel without toothpaste, but subsequent brushing with toothpaste would weaken the enamel [79].

Toothbrushing removes more of the tooth structure than ultrasonication, as the loss of softened enamel increases with the increased brush strokes [80], reducing surface microhardness [81,82]. Similarly, brushing with fluoride containing toothpastes alters the surface enamel [83] but with fewer microsurface changes than toothpastes with probiotics or xylitol [84].

Whitening toothpaste, often referred to as lightening toothpaste, reduces the shade of the tooth by one color [85]. Blue covarin is an effective tooth-whitening agent [86]. Although a few studies have shown no difference in the abrasion caused by whitening or conventional toothpaste [87,88], some reports suggest the active ingredients in whitening toothpaste result in an increased depth of dentin and enamel abrasion [89]. The roughness of the surface increases without affecting the whitening of the tooth [90]. Whitening toothpastes loaded with diamond powder show a U-shaped loss of dentin [91]. Charcoal-containing whitening toothpaste shows the highest abrasive wear [92,93,94], with the RDA and REA values of charcoal toothpaste varying from 24 to 166 and 0 to 14. Moreover, these toothpastes lack the beneficial effect of fluoride on oral health [95]. High-risk cervical abrasion/erosion patients should avoid these [96].

Dentinal hypersensitivity-related toothpaste formulations contain ingredients capable of occluding tubules [97]. Yet, some of their ingredients, silica, calcium, and sodium-phosphate, aggravated tooth abrasion [98,99]. Calcium silicate and NaPO4 toothpaste did not reduce enamel/dentin loss compared to non-fluoridated pastes [100]. Higher brushing forces were needed to achieve a larger area of tubule occlusion with NovaMin^®^, (GlaxoSmithKline, London, UK) a desensitizing agent. Encouraging reports about Colgate-sensitive pro-relief or calcium sodium phosphosilicate showed no increase in surface loss.

Joao Souza [101] concluded that tooth loss was greater if toothpastes had lower pH, lower fluoride and calcium concentrations (Ca^2+^), higher PO_4_^3−^ concentration, smaller particles sizes, higher % weight of solid particles, and lower wettability. Brushing with either a liquid toothpaste (RDA) or water and no toothpaste caused minimal enamel and dentin loss [102] and could be recommended for patients with cervical abrasions to avoid further destruction of the tooth structure.

## 6. Management

**Figure 3:**
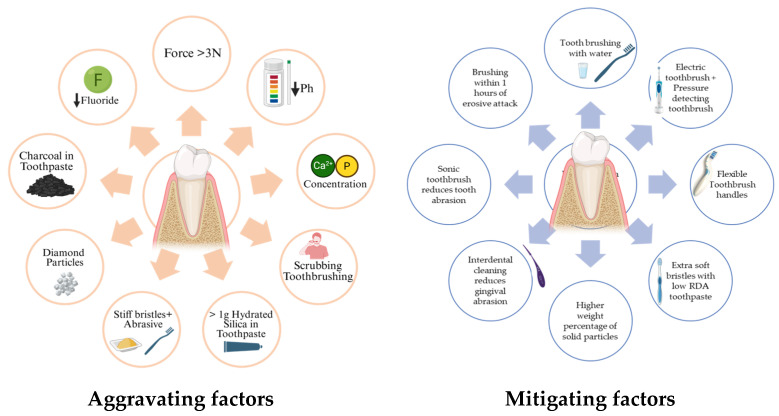
Factors that aggravate and mitigate tooth surface loss.

Gingival trauma can occur due to overzealous brushing over the gingiva margins, manifesting as blanched hyperkeratotic areas and as gingival recession. Many non-surgical and surgical treatment modalities are available to correct gingival recession. However, changing the technique/type of brush/toothpaste before advising any surgical therapy is paramount (Table 3). An individual must learn and practice an apt brushing technique with optimal brushing pressure to maintain oral hygiene and prevent soft tissue detriment (Figure 3). Rubber-bristled interdental cleaners favor interdental cleaning, promoting better plaque control and thereby withstanding the effects of trauma over areas with gingival inflammation [103,104]. While non-surgical options help prevent plaque accumulation, correcting established wedge-shaped/saucer-shaped defects requires restorations and surgical options such as soft tissue graft procedures.

Some respite exists in the fact that abrasion induced by two minutes of brushing needs 24 h to remineralize completely [105]. The abrasion resistance of enamel improves if brushing is delayed by an hour following an erosive attack [106,107]. A standard manual or sonic toothbrush with appropriate technique can be encouraged for progressive abrasion [4]. Poor oral hygiene maintenance during orthodontic treatment promotes white spot lesions, which can be managed with correct brushing techniques at regular intervals using a low-abrasive toothpaste with remineralizing agents [108].

**Figure 4:**
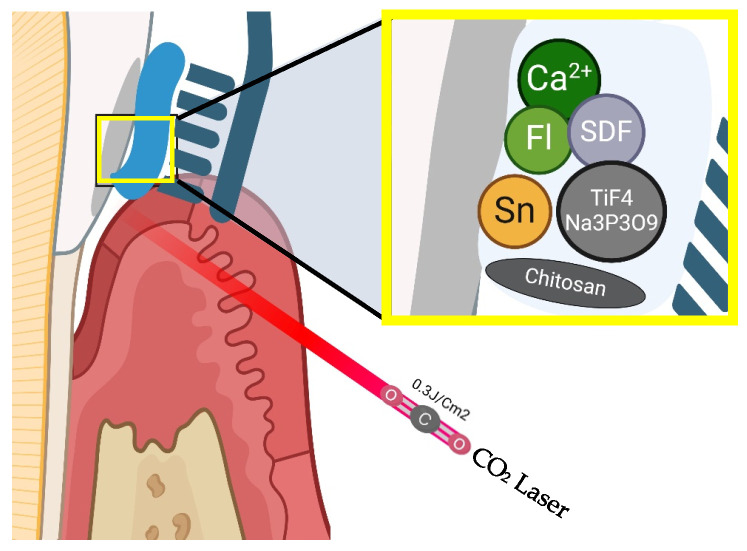
Tooth surface remineralization.

Fluoride (amine or sodium forms), tin (stannous fluoride/stannous chloride), calcium carbonate, and chitosan toothpastes, as well as bioactive glass-containing toothpastes, significantly reduce erosion and abrasion [69,109,110,111,112,113]. Sodium trimetaphosphate in fluoride varnish [114], rinsing with an iron solution [115], and carbon dioxide laser irradiation (0.3 J/cm^2^; 226 Hz) (Figure 4) [116] also mitigate erosion/abrasion.

**Table 3:** Systematic reviews and randomized controlled trials on toothbrush type/technique and toothpastes.

Author and Year	Aim	Study Type, Sample Size	Outcome
Systematic reviews			
Tomás, D.B.M. et al. (2023) [117]	Qualitative synthesis of the available literature on the use of activated charcoal-based toothpaste for tooth whitening.	Out of 208 articles, 11 met the inclusion criteria, the risk of bias of the selected studies was determined as medium–high.	Most studies agree that activated charcoal toothpaste has a higher abrasive potential.
Van der Weijden, F et al. (2022) [103]	Efficacy of a rubber-bristled interdental cleaner (RBIC) as an adjunct to toothbrushing (TB) compared to other interdental cleaning devices on plaque and gingivitis parameters.	The search retrieved 142 unique papers; 6 studies with 10 comparisons were included in a descriptive analysis. Five RCTs compared RBICs with interdental brushes (IDBs), four with dental floss (DF) and one with manual TBs only.	A weak certainty exists that a RBIC is indicated for gingivitis and plaque reduction.
Jamwal, N et al. (2022) [85]	The relationship between whitening toothpastes and surface roughness as well as the microhardness of human teeth.	A total of 125 articles were obtained through a key word search. After duplicate removal and title screening, 17 articles were eligible for full-text review. Finally, 7 studies were included for systematic review, and meta-analysis was conducted on 4 studies.	Whitening toothpaste lightens the tooth color but can cause increases in surface microroughness.
Ranzan, N et al. (2019) [42]	To examine soft tissue lesions caused by different bristle stiffnesses and bristle end shapes in manual toothbrushes in adult individuals.	Thirteen studies were included from 1945 initially retrieved papers. The toothbrush bristle end shape was investigated in six studies and bristle stiffness in two, and both features were investigated in five studies.	Soft and extra-soft toothbrushes tend to be safer. Oral lesions are similar in tapered and round-ended bristles.
Muller-Bolla, M.; Courson, F. (2013) [24]	The most effective technique of toothbrushing in children.	The level of evidence was moderate and 6 out of 534 articles were included.	The horizontal technique was the most effective up to 6–7 years of age.
Randomized clinical trials			
Sutor, S et al. (2025) [45]	Powered toothbrush (PT) on the size and number of pre-existing gingival recessions (GR) in comparison to a manual toothbrush (MT).	Prospective, single-blind, parallel-group, randomized controlled clinical study. In total, 87 out of 92 participants completed the study (MT/PT: n = 42/n = 45). GR ≥ 2 mm; twice daily brushing with a manual or power toothbrush. Primary outcome–mean change in GR over 36 months.	PT seems to be favorable in patients with pre exiting gingival recession.
Hennequin-Hoenderdos, N.L et al. (2018) [104]	Efficacy of a rubber-bristled interdental cleaner (RBIC) compared to an interdental brush (IDB) in reducing gingivitis.	Two-treatment, parallel, split-mouth, examiner-blind RCT, evaluating the reversal of experimental gingivitis in 46 healthy patients.	A RBIC, in conjunction with manual toothbrushing, was found to be more effective in reducing gingival inflammation after 4 weeks. The RBIC caused less abrasion of the gingiva and was appreciated more by the participants.
Graetz, C et al. (2017) [46]	Link between bristle splaying and gingival recession.	A parallel-group, randomized, controlled, clinical trial, With 110 healthy participants, pre-existing gingival recessions, and a 12-month duration; manual (MT) vs. powered toothbrush (PT). Wear was measured using the Bristle Splaying Index (BSI).	After 3 months, the PT group demonstrated a lower BSI than the MT group. Greater bristle splaying was associated with a higher risk of increase in GR in subjects using a MT but not a PT.
N L -Hoenderdos et al. (2017) [118]	Filament end-rounding and gingival abrasion.	Crossover, split-mouth, contralateral, double-blinded, randomized design with 46 healthy participants. Oral soft tissue and gingival abrasion were assessed.	If the brushes have 40–50% of bristles with end roundedness it can provide greater reduction in gingival abrasion.
Dörfer, C.E et al. (2016) [47]	Oscillating–rotating power toothbrush or an ADA reference manual toothbrush on pre-existing gingival recession.	Controlled, prospective, single-blind, parallel-group study; healthy subjects with pre-existing recession were randomized and brushed with a power toothbrush (n = 55) or an ADA reference manual toothbrush (n = 54) for a 3-year study period.	Recession significantly reduced after 3 years of brushing with either of the toothbrushes.
Sälzer, S et al. (2016) [48]	Effect of brushing with either a multidirectional PT or American Dental Association reference manual toothbrush (MT) on mid-buccal preexisting GR (PreGR).	12-month prospective, single-masked, parallel-group, randomized, controlled clinical study. 107 participants without periodontitis with at least two teeth showing PreGR ≥ 2 mm were randomized to a group brushing with either an manual or powered toothbrush.	Neither the PT nor MT led to an increase in PreGR during 12 months of daily use.
Greggianin, B.F et al. (2013) [43]	Incidence of gingival fissures after the use of soft and medium–hard toothbrushes.	35 participants (14–20 years old), with periodontal attachment loss (PAL) < 1 mm, were assigned to soft or medium–hard toothbrushes in a crossover design with a wash-out of 10 days between two 28-day periods.	Gingival fissures are a common feature associated with toothbrushing. Medium–hard toothbrushes, male gender, time, and previous PAL are significant risk factors for the incidence of gingival fissures.

## 7. Conclusions

Brushing as a routine oral hygiene measure depends on correct execution for good oral health. The variety of oral care products available can cloud subject judgment. Dental professionals can recommend products and techniques specific to an individual’s condition and dexterity, promoting the most suitable oral hygiene routine for them. Furthermore, policymakers can insist on compulsory indication of the RDA values on the toothpaste packets for public awareness and informed decision-making. The review provides insight into all the measurable aspects of a toothbrush and the associated aids to help clinicians select appropriate aid and allow researchers to explore and improve them for the benefit of patients.

## Abbreviations

The following abbreviations are used in this manuscript:

PTB	Polybutylene terephthalate
ETW	Erosive tooth wear
REA	Relative Enamel Abrasivity
RDA	Relative Dentin Abrasivity
ADA	American Dental Association
RBIC	Rubber-bristled interdental cleaner
IDB	Interdental brush
